# Therapeutic efficacy of the optimization of thyroid function, thrombophilia, immunity and uterine milieu (OPTIMUM) treatment strategy on pregnancy outcomes after single euploid blastocyst transfer in advanced age women with recurrent reproductive failure

**DOI:** 10.1002/rmb2.12554

**Published:** 2023-12-22

**Authors:** Keiji Kuroda, Takashi Horikawa, Azusa Moriyama, Yuko Ojiro, Satoru Takamizawa, Hideaki Watanabe, Tetsuo Maruyama, Shuko Nojiri, Koji Nakagawa, Rikikazu Sugiyama

**Affiliations:** ^1^ Center for Reproductive Medicine and Endoscopy Sugiyama Clinic Marunouchi Tokyo Japan; ^2^ Department of Obstetrics and Gynecology Juntendo University Faculty of Medicine Tokyo Japan; ^3^ Center for Reproductive Medicine and Implantation Research Sugiyama Clinic Shinjuku Tokyo Japan; ^4^ Medical Technology Innovation Center Juntendo University Tokyo Japan; ^5^ Clinical Research and Trial Center Juntendo University Hospital Tokyo Japan

**Keywords:** infertility, IVF, preimplantation genetic testing for aneuploidy, recurrent implantation failure, recurrent pregnancy loss

## Abstract

**Purpose:**

To clarify the efficacy of the OPtimization of Thyroid function, Thrombophilia, IMmunity and Uterine Milieu (OPTIMUM) treatment strategy on pregnancy outcomes after euploid blastocyst transfer in advanced age women with recurrent implantation failure (RIF) and/or recurrent pregnancy loss (RPL).

**Methods:**

Between January 2019 and May 2022, 193 consecutive women aged ≥40 years with RIF and/or RPL received single euploid blastocyst transfer. Before embryo transfer, 127 women underwent RIF/RPL testing. Chronic endometritis was treated with mainly antibiotics, aberrant high Th1/Th2 cell ratios with vitamin D and/or tacrolimus, overt/subclinical hypothyroidism with levothyroxine, and thrombophilia with low‐dose aspirin. We compared pregnancy outcomes in the women who did and did not receive the OPTIMUM treatment strategy.

**Results:**

Women with RIF/RPL in the OPTIMUM group had significantly higher clinical pregnancy and livebirth rates than did those in the control group (clinical pregnancy rate of 71.7% and 45.5%, *p* < 0.001; livebirth rate of 64.6% and 39.4%, *p* = 0.001, respectively). However, preimplantation genetic testing for aneuploidy with and without OPTIMUM promoted low miscarriage rates with no significant difference between them (9.9%, and 13.3%, respectively; *p* = 0.73).

**Conclusions:**

The OPTIMUM treatment strategy improved clinical pregnancy rates after single euploid blastocyst transfer; but not miscarriage rates.

## Graphical abstract

Pregnancy outcomes according to age after single euploid blastocyst transfer in women who underwent the Optimization of Thyroid function, Thrombophilia, Immunity and Uterine Milieu (OPTIMUM) treatment strategy or did those who did not (control) are shown. Among women aged 40 years in the OPTIMUM group had significantly higher clinical pregnancy rates than those in the control group. Women aged 41 years in the OPTIMUM group had also relatively higher clinical pregnancy rate, compared to those in the control; however, no significant differences were noted among women aged ≥42 years.

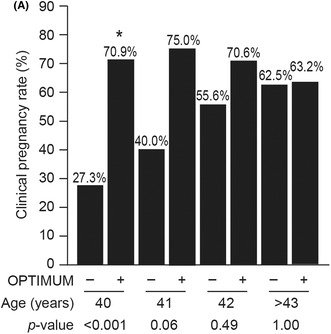

## 1 INTRODUCTION

Implantation failure and pregnancy loss are mainly caused by embryonic chromosomal abnormalities.^1^, ^2^ Therefore, patients with a history of recurrent implantation failure (RIF) and recurrent pregnancy loss (RPL) may suffer from repeated sporadic and fortuitous reproductive failure. However, aside from embryonic factors, various other factors, such as immune, endocrine, microbial, anatomic, hematologic, genetic, and lifestyle factors, can intricately interfere with the processes associated with embryo implantation and maintenance of pregnancy, leading to multiple instances of reproductive failure.^3^, ^4^, ^5^, ^6^, ^7^

We had previously reported on a combination treatment for abnormalities in the intrauterine environment, immunological status, thyroid function, and blood coagulation (i.e., thrombophilia), referred to as the OPtimization of Thyroid function, Thrombophilia, IMmunity and Uterine Milieu (OPTIMUM) treatment strategy, to address RIF and RPL.^8^, ^9^ In this strategy, risk factors for implantation failure and miscarriage are simply detected using minimum variable tests and are treated. This approach can treat non‐embryonic risk factors for RIF and RPL without high‐cost examinations, including preimplantation genetic screening for aneuploidy (PGT‐A), endometrial receptivity analysis (ERA), and endometrial microbiome testing. In European Society of Human Reproduction and Embryology (ESHRE) guideline for RIF, the OPTIMUM treatment strategy is introduced as treatment based on diagnostic findings.^10^ Among women aged <40 years, the OPTIMUM treatment strategy promoted childbearing in 72.7% of those with RIF (3–14 instances of implantation failure) within two embryo transfer (ET) cycles^8^ and 78.1% of those with RPL (2–5 pregnancy losses) at the first pregnancy after this treatment.^9^ Those who received the OPTIMUM treatment strategy showed significantly much better pregnancy outcomes than did those who did not. However, studies on both RIF and RPL found no significant differences in pregnancy outcomes between women over 40 years who did and did not receive the OPTIMUM treatment strategy.^8^, ^9^

Aneuploidy rates of embryos increase with female aging, resulting in decreased embryo implantation rates and increased pregnancy loss rates.^11^, ^12^, ^13^ According to the results of PGT‐A in the United States, women aged 40, 42, and 44 years had blastocyst euploidy rates of 41.8%, 24.9%, and 11.8%, respectively.^14^ Therefore, the treatment of detected risk factors for reproductive failure using the OPTIMUM treatment strategy could not significantly improve the pregnancy outcomes in advanced age women with high embryonic aneuploidy rates. To shorten the time to childbearing among advanced age women with a history of multiple instances of reproductive failure, selection of an euploid blastocyst using PGT‐A is needed.

The current study analyzed the pregnancy outcomes and therapeutic efficacy of the OPTIMUM treatment strategy in women aged ≥40 years with a history of RIF and/or RPL who underwent ET using a single elective euploid blastocyst.

## 2 MATERIALS AND METHODS

### 2.1 Participant selection

This observational cross‐sectional study included a total of 201 consecutive women with a history of RIF and/or RPL who underwent single euploid blastocyst transfer (SEBT) at our clinic between January 2019 and May 2022. RIF was defined as implantation failure after three or more ET cycles using 5–6 days after fertilization of morphologically good blastocysts without grade C blastocysts in both inner cell mass and trophectoderm based on the Gardner classification as reported previously.^8^ RPL was defined as two or more consecutive clinical pregnancy losses based on global guidelines for RPL.^15^, ^16^ Notably, pregnancy losses in this study did not include biochemical and ectopic pregnancies. Among the recruited women, 127 underwent our RIF/RPL testing to detect risk factors for reproductive failure (OPTIMUM group). Eight women who did not wish to undergo a part of RIF/RPL testing were excluded. The other 66 women who were not offered or did not desire RIF/RPL testing were also recruited as the control group. In the OPTIMUM group, 96 and 53 women experienced RIF and RPL, respectively, whereas 22 women had a history of both RIF and RPL.

Pregnancy prognosis of women with RIF and/or RPL in the OPTIMUM group was then compared to that of the control group. Human chorionic gonadotropin (hCG) positive was defined when hCG levels were >5 mIU/mL at 8 to 10 days after ET. Clinical pregnancy was diagnosed based on the presence of an intrauterine gestational sac via transvaginal ultrasound. Pregnancy loss was defined as a loss in clinical pregnancy, not biochemical pregnancy. Infertility was diagnosed based on failure to achieve clinical pregnancy after unprotected intercourse for 1 year or longer.

To analyze factors predicting outcomes after the OPTIMUM treatment strategy, we also compared women who had a successful live birth (success group) and those who experienced implantation failure or pregnancy loss (failure group) on their first SEBT after the OPTIMUM. This study was approved by the local ethics committee of Sugiyama Clinic (No. 18–002 and 22–008).

### 2.2 Protocol of the OPTIMUM treatment strategy

**FIGURE 1:**
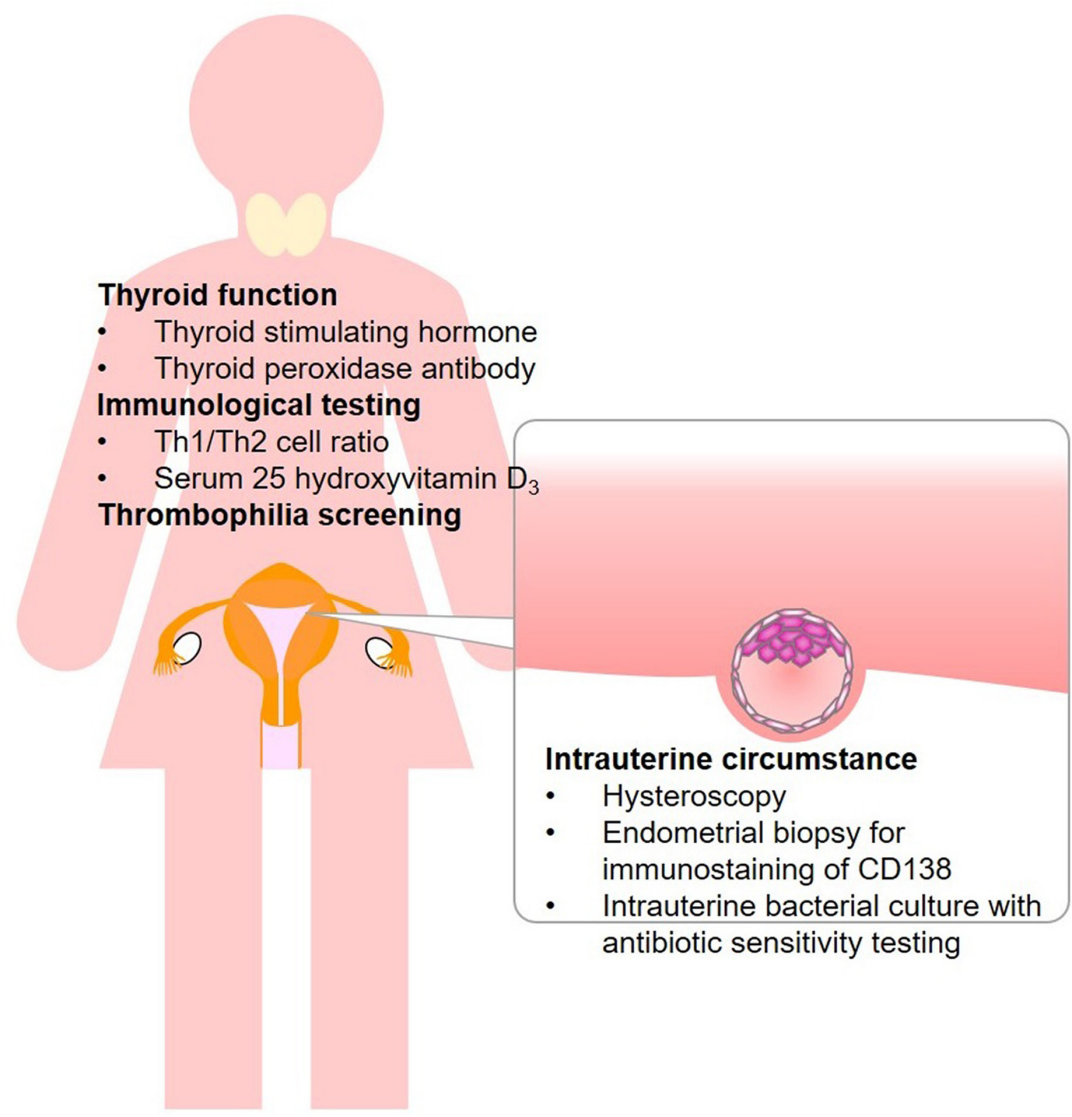
RIF/RPL testing. Recurrent implantation failure (RIF)/recurrent pregnancy loss (RPL) testing consists of local testing for intrauterine circumstances including a hysteroscopy; endometrial biopsy for CD138 immunostaining and bacterial culture; and systemic testing for the levels of 25‐hydroxyvitamin D_3_ and IFN‐γ and IL‐4‐producing helper T cells (Th1 and Th2 cells), thyroid function, and thrombophilia.

The design of the OPTIMUM treatment strategy has been described previously.^8^, ^9^ Our RIF/RPL testing consisted of the following (Figure 1): local testing for intrauterine circumstances including hysteroscopic examination, endometrial biopsy for CD138 immunohistochemistry staining, and intrauterine bacterial culture; and systemic testing such as immunological testing including measurement of serum levels of 25‐hydroxyvitamin D_3_ (25OHVD) and interferon (IFN)‐γ and interleukin (IL)‐4–producing helper T (Th) cells (Th1 and Th2 cells), testing for thyroid function including thyroid‐stimulating hormone (TSH) and thyroid peroxidase antibody (TPOAb) levels, and thrombophilia screening including measurement of lupus anticoagulant, anticardiolipin antibody, and anti‐β2‐GP1 antibody levels, protein C and S activities, and factor XII levels.

**FIGURE 2:**
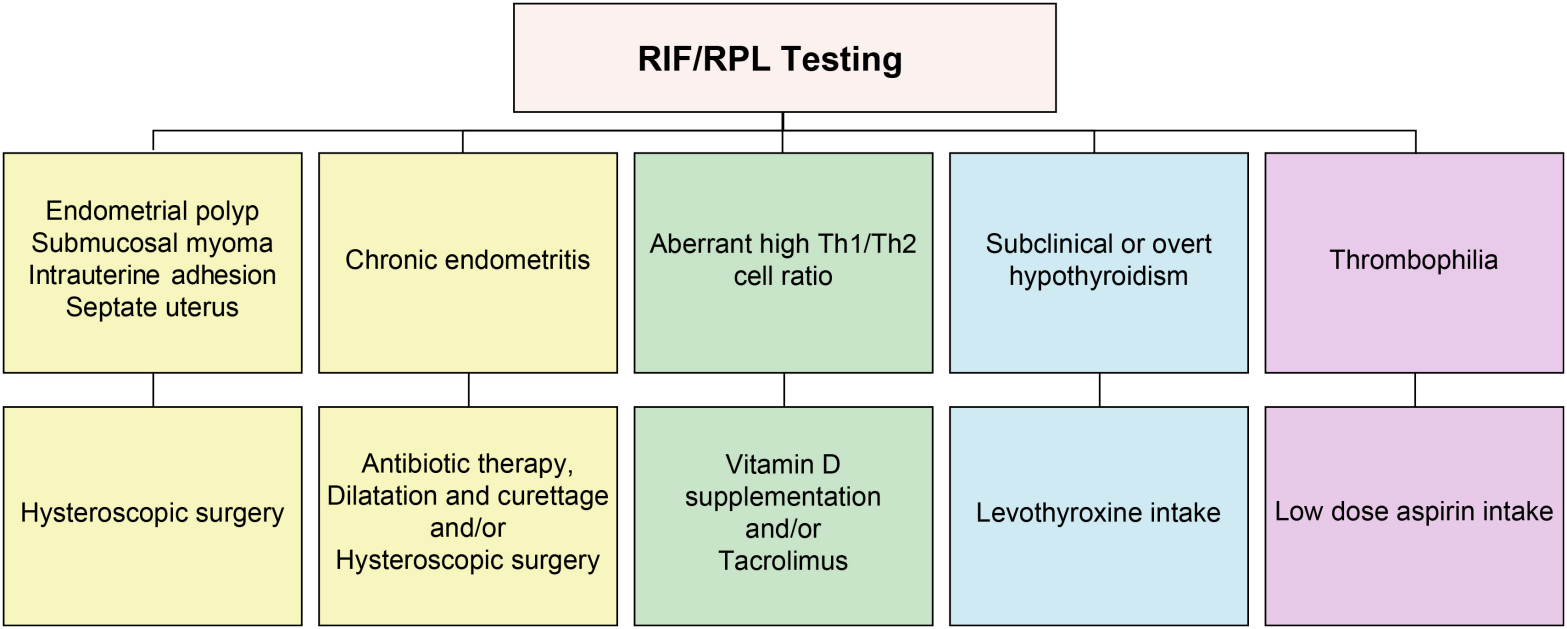
Therapeutic protocol in the OPTIMUM treatment strategy. Intrauterine abnormalities were treated with hysteroscopic surgery, chronic endometritis with antibiotics, dilation and curettage, and/or hysteroscopic surgery, high Th1/Th2 cell ratios with vitamin D supplementation and/or tacrolimus, hypothyroidism with levothyroxine, and thrombophilia with low‐dose aspirin.

**FIGURE 3:**
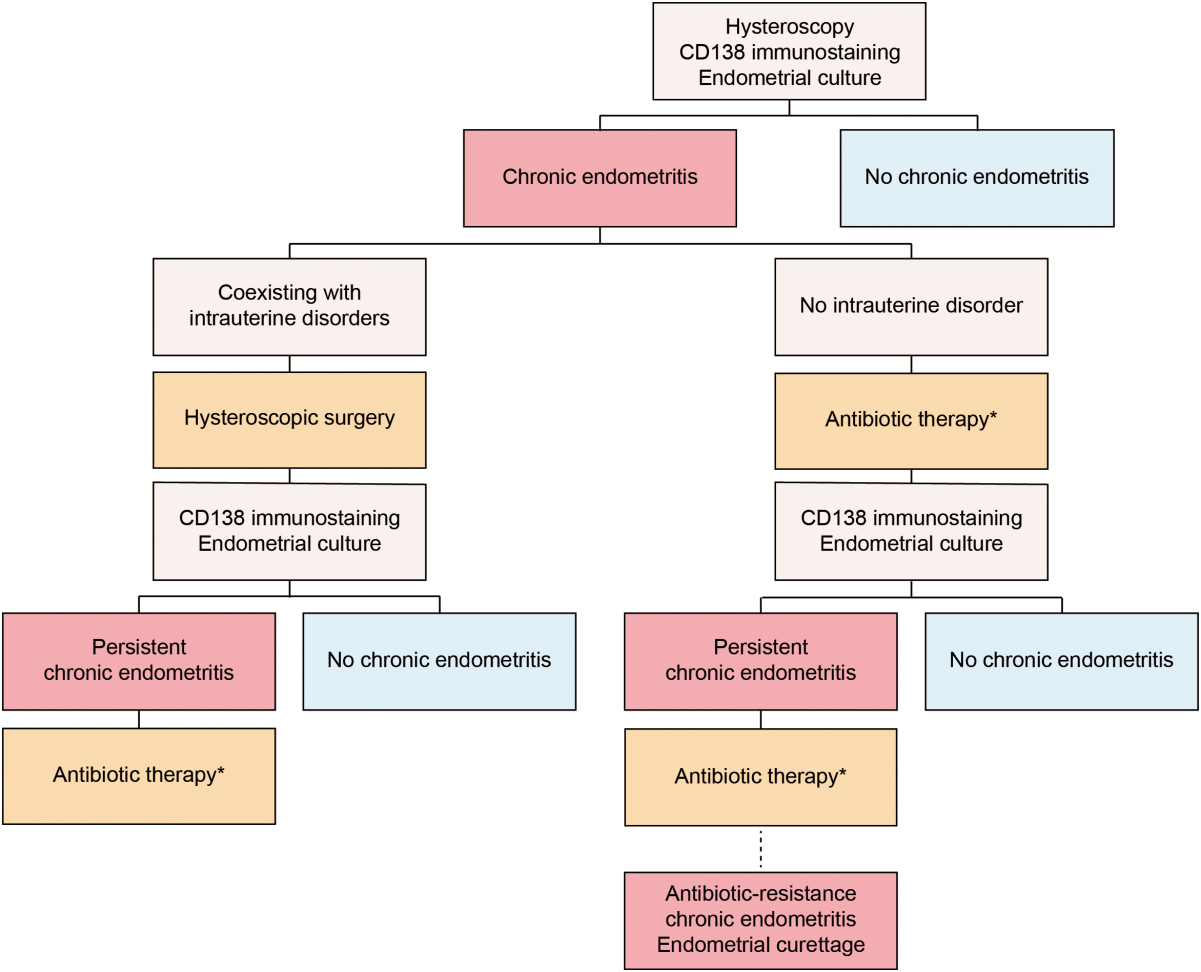
Treatment protocol of chronic endometritis. When organic lesions are found via hysteroscopy, firstly hysteroscopic surgery is performed. When chronic endometritis (CE) is diagnosed from the specimen, CD138 immunostaining and bacterial culturing tests are reexamined without antibiotic use in the subsequent menstruation cycle after surgery. When CE is detected in the patients without intrauterine lesions, oral antibiotics for 2 weeks are administered. If CE was not disappeared after two or more cycles of antibiotic use, gentle endometrial curettage is performed.

The therapeutic protocol in the OPTIMUM treatment strategy is shown in Figure 2. And, the treatment protocol of intrauterine abnormalities is summarized in Figure 3. When intrauterine organic lesions were found in hysteroscopic examination, hysteroscopic surgery was initially performed. During hysteroscopic surgery, all intrauterine abnormalities and typical chronic endometritis (CE) findings^17^ were removed using a monopolar resecting loop (Olympus) without applying electrodes, as described previously.^18^ The specimens were sent to BML, Inc. for both endometrial CD138 immunostaining and bacterial culturing tests. Pathologists stained the specimens using anti‐CD138 antibodies (M7228; Dako, Agilent Technologies, Ltd., United States) and counted the CD138‐positive cells (BML, Inc.). In this study, CE was diagnosed based on the presence of ≥5 CD138‐positive plasmacytes in 10 nonoverlapping random stromal areas at 400‐fold magnification. Most CE with intrauterine organic lesions can be cured by hysteroscopic surgery without doxycycline use.^18^, ^19^ Therefore, we did not use unnecessary antibiotic therapy after surgery. And we re‐examined endometrial CD138 immunostaining and bacterial culturing tests using endometrial suction curette (Pipet Curet; Fuji Medical Corporation, Tokyo, Japan) during the luteal phase in the subsequent menstruation cycle after surgery.

Patients diagnosed with CE without intrauterine disorders received oral bacterium‐sensitive antibiotics for 2 weeks or doxycycline (Vibramycin® tablets; Pfizer Japan Inc., Tokyo, Japan) at 100 mg twice a day for 2 weeks, based on the results of endometrial bacterial culture tests with or without specific bacteria except for *Lactobacillus* spp. or *Bifidobacterium* spp., respectively. If CE persisted with or without specific bacteria, bacterium‐sensitive antibiotics for 2 weeks or ciprofloxacin (Ciproxan®, 200 mg; Bayer Yakuhin, Ltd., Osaka, Japan) and metronidazole (Flagyl®, 250 mg; Shionogi & Co., Ltd., Osaka, Japan) twice daily for 2 weeks were administered as second‐line therapy. If CE persisted after two or more cycles of antibiotic therapy, gentle dilation and curettage was performed for the artificial removal of the inflamed endometrium.

Immunological testing consisted of measuring serum levels of 25OHVD, IFN‐γ, and IL‐4 using SRL Inc.^20^, ^21^ Vitamin D supplementation was provided at 25 or 50 μg daily for patients with 25OHVD levels ≥20 and <30 ng/mL or <20 ng/mL, respectively, based on our previous report.^20^ Aberrantly high Th1 cell levels and Th1/Th2 cell ratios were defined as ≥28.8 and ≥11.8, respectively, based on data from normal fertile women and previous reports.^22^, ^23^ Given that vitamin D can play a role in suppressing elevated Th1 cell levels and Th1/Th2 cell ratios,^20^ women with aberrantly high Th1 cell levels or Th1/Th2 cell ratios and low 25OHVD levels were re‐examined for serum levels of 25OHVD and Th1 and Th2 cells after ≥2 months of vitamin D supplementation. When high Th1/Th2 cell ratios could not be appropriately suppressed by supplementation, an immunosuppressive drug, tacrolimus (Prograf® capsules, 1 mg, Astellas Pharma), was administered as described previously.^21^, ^23^ Women with Th1/Th2 cell ratios of 11.8–18.9 and ≥19.0 were treated with 2 and 3 mg of tacrolimus daily, respectively, from 1 day before ET for those with RIF and from the day a positive pregnancy test was confirmed (4–5 weeks of gestation) for those with RPL. In women with aberrantly elevated Th1 cell levels (i.e., ≥28.8), the dosage of tacrolimus was increased by an additional 1 mg.^23^

For thyroid function, the threshold at which thyroid abnormalities were treated using levothyroxine among the included women was a TSH level of 2.5–4.23 μIU/mL and TPOAb positivity or a TSH level of >4.23 μIU/mL. All women receiving levothyroxine therapy maintained TSH levels of <2.5 μIU/mL and continued until live birth.

Women who tested positive for thrombophilia received low‐dose aspirin (Bafferin Combination Tablet A81, 81 mg, Eisai Co., Ltd.) 5 days after ET for those with RIF and the day a positive pregnancy test was confirmed for those with RPL. Low‐molecular‐weight heparin was not administered throughout this study.

When none of the risk factors, except for vitamin D insufficiency, were detected in patients with RPL, we recommended progesterone supplementation with 30 mg of dydrogesterone tablets (Dufaston® Tablets 5 mg; Mylan EPD LLC.) 3 times daily or 90 mg of OneCrinone® (Vaginal progesterone gel; Merck Biopharma Co., Ltd.) once daily until 12 weeks of gestation. Progesterone is essential for maintaining pregnancy; therefore, progesterone supplementation has potential therapeutic efficacy in preventing spontaneous abortion among patients with unexplained RPL as described in previous trials.^24^, ^25^

### 2.3 Medical interview and lifestyle modification

We determined the lifestyle habits of women with RPL and recommended modifications, such as cessation of smoking and caffeine and alcoholic beverage consumption during pregnancy, as well as exercise and dietary modifications for obesity. Considering the association between maternal phycological stress and progressive risk of pregnancy loss,^26^, ^27^ counseling was provided to reduce maternal stress and anxiety as necessary.

### 2.4 PGT‐A and single euploid blastocyst transfer

Flexible progestin‐primed ovarian stimulation was mainly used for ovarian stimulation approaches in this study. Patients received 50 mg of clomiphene citrate (Clomid®, Fuji Pharma) or 2.5 mg of letrozole (Nippon Kayaku Co., Ltd.) once daily for 5 days starting on day 3 of the menstruation cycle and 150–225 IU of recombinant follicle‐stimulating hormone (Gonal‐f®, Merck) or human menopausal gonadotropin (HMG Ferring, Ferring Pharmaceuticals) administered on cycle days 3, 4, 6, and 8 or on consecutive days starting on day 3 of menstruation cycle depending on the ovarian reserve status and previous ART data of the patients. On days 6–8, 4 mg of chlormadinone acetate (Lutoral® tablets; Fuji Pharma) twice daily or 10 mg of medroxyprogesterone acetate (Hysron® tablets 5; Kyowa Kirin Co., Ltd.) was also started until ovulation was induced. When dominant follicles ≥17 mm were confirmed, 250 μg of a recombinant hCG (Ovidrel®; Merck Biopharma Co., Ltd.) injection and/or 600 μg of a nasal buserelin acetate spray (Buserecur®; Fuji Pharma) was administered to induce ovulation. After 35–36 h, we aspirated the follicles transvaginally and performed conventional IVF or intracytoplasmic sperm injection (ICSI) depending on spermatic findings and previous fertilization rates. When expanded blastocysts (grade 4 or more of the Gardner classification) at 5–6 days after fertilization were recognized, 5–10 cells were biopsied from the trophectoderms of the blastocysts and sent to Igenomix Japan, K.K. (Tokyo, Japan) for PGT‐A. The blastocysts were cryopreserved once. The PGT‐A protocol has been described previously.^28^ Euploidy was defined as mosaicism with <30% of the biopsied cells.

To maximize embryo competency during the ET cycle, all women in this study underwent single vitrified‐warmed blastocyst transfer with laser‐assisted hatching under spontaneous ovulation or hormonal replacement cycle. Although the OPTIMUM treatment strategy did not include endometrial receptivity testing, 25 women underwent ERA (Igenomix Japan, K.K.) or ERPeak^SM^ (CooperSurgical, Inc, United States) in the OPTIMUM group upon their request after CE was not detected given the potential influence CE on the window of implantation.^29^, ^30^ Thereafter, personalized ET was performed based on the results.

### 2.5 Statistical analysis

All statistical analyses were performed using Statistical Analysis System version 9.4 (SAS Institute, Cary, NC, United States). Significant differences in continuous variables were analyzed using the *t*‐test, whereas significant differences in categorical variables were analyzed using the chi‐square and Fisher's exact tests as appropriate. To identify factors independently predicting pregnancy outcomes of SEBT after the OPTIMUM treatment strategy in women with RIF/RPL, a multivariable logistic regression model was established while controlling for confounders. Independent variables included age, prevalence of RIF, prevalence of RPL, embryo quality (reference: morphologically good blastocyst), culture time until expanded blastocyst (reference: 5 days), impaired intrauterine circumstance, aberrantly high Th1 cell levels and/or Th1/Th2 cell ratios, thyroid disorder, and thrombophilia. Odds ratios (ORs) and their 95% confidence intervals (CIs) were computed. The level of significance was set at *p* < 0.05.

## 3 RESULTS

### 3.1 Prevalence of risk factors for implantation failure and pregnancy loss

Table 1 summarizes the characteristics of the women with RIF/RPL. Women with RIF and RPL experienced 4^3^, ^4^, ^5^, ^6^, ^7^, ^8^, ^9^, ^10^, ^11^, ^12^, ^13^, ^14^, ^15^ previous ET cycles and 2^2^, ^3^, ^4^, ^5^ times of pregnancy losses, respectively. The OPTIMUM group had a significantly higher and lower prevalence of RIF and RPL than did the control group, respectively. The prevalence of risk factors for implantation failure and pregnancy loss are presented in Table S1. The prevalence of intrauterine abnormalities was 56.8% (42 women), 48.4% (15 women), and 50.0% (11 women) among women with a history of RIF only, RPL only, and both RIF and RPL, respectively. Among the 68 women with intrauterine abnormalities, 58 (85.3%) had CE, the most prevalent abnormality. Regarding immunological status, 63.8% of the women with RIF and/or RPL had vitamin D insufficiency or deficiency. The prevalence of an aberrantly elevated Th1/Th2 cell ratio was 27.0% (20 women), 25.8% (8 women), and 45.5% (10 women) among women with RIF, RPL, and RIF + RPL, respectively. Subclinical or overt hypothyroidism was detected in 17.6% (13 women), 6.5% (2 women), 18.2% (4 women) of the women with RIF, RPL, and RIF + RPL, respectively. In addition, thrombophilia was recognized in 18.9% (14 women), 16.1% (5 women), and 22.7% (5 women) of the women with RIF, RPL, and RIF + RPL, respectively. RIF/RPL testing showed that 33 (44.6%), 7 (22.6%), and 10 (45.5%) of the women had ≥2 risk factors; however, 20 (27.0%), 8 (25.8%), and 31 (13.6%) of those with RIF, RPL, and RIF + RPL had no risk factors, respectively.

**TABLE 1:** Clinical characteristics and pregnancy outcomes in control and OPTIMUM groups.

	Control, *n* = 66	OPTIMUM, *n* = 127	*p*‐Value
Age, years	41.5 ± 1.4 (40–45)	41.1 ± 1.3 (40–46)	0.08
Anti‐Müllerian hormone, ng/mL	2.1 ± 1.3	2.5 ± 1.7	0.44
Pregnancy history			
Gravida	3 (0–7)	2 (0–6)	0.005*
Parity	0 (0–4)	0 (0–2)	0.04*
No. of previous miscarriages	2 (0–4)	1 (0–5)	0.10
Prevalence of RPL	42 (63.6)	53 (41.7)	0.004*
Previous ART treatment			
No. of previous embryo transfer cycles	3 (0–13)	4 (0–13)	0.21
Prevalence of RIF	30 (45.5)	96 (75.6)	<0.001*

*Note*: Data are presented as mean ± SD (range) or mean ± SD or median (range) or *n* (%).

Abbreviations: ART, assisted reproductive technology; OPTIMUM, optimization of thyroid function, thrombophilia, immunity and uterine milieu; RIF, repeated implantation failure; RPL, recurrent pregnancy loss.

* *p* < 0.05.

### 3.2 Treatment for impaired intrauterine circumstances and aberrantly high Th1/Th2 cell ratios

Among the 68 women with impaired intrauterine circumstances, 19 with intrauterine disorders underwent hysteroscopic surgery. CE was recognized in 9 women after surgery, and recovery from CE was confirmed in 7 women (77.8%) at re‐examination of CD138 immunostaining in the subsequent cycle after surgery without additional antibiotic therapy. In the remaining 2 women (28.6%), CE was treated using bacterium‐sensitive antibiotics based on the results of the endometrial bacterial culture test. Among the 49 women who had CE without intrauterine disorders, 34 (69.4%) and 14 (28.6%) were successfully cured from CE after the first and second cycles of antibiotic therapy, respectively. One woman (2.0%) with persistent CE after 2 cycles of antibiotic therapy underwent dilation and curettage, leading to recovery from CE. Finally, all women included in this study were cured of CE.

Among the 38 women with aberrantly high Th1 cell levels (≥28.8) and/or Th1/Th2 cell ratios (≥11.8), 21 (55.3%) had insufficient vitamin D levels. Among the 20 women who underwent Th1/Th2 cell ratio re‐examination 2–5 months after starting 25 or 50 μg of daily vitamin D supplementation, 13 (65.0%) were able to normalize their Th1 cell levels and Th1/Th2 cell ratios. For the 25 women with an elevated Th1/Th2 cell ratio even after vitamin D intake, daily use of tacrolimus 1 day before ET in those with RIF and after a positive pregnancy test in those with RPL is recommended. No side effects of vitamin D and tacrolimus therapy were noted.

### 3.3 Pregnancy outcomes following single euploid blastocyst transfer after the OPTIMUM treatment strategy

To determine the therapeutic efficacy of the OPTIMUM treatment strategy for improving pregnancy outcomes in advanced age women with a history of RIF and/or RPL, pregnancy outcomes in those who did and did not receive the OPTIMUM treatment strategy were analyzed (Table 2). The number of biopsied blastocysts until euploid embryos were detected in the OPTIMUM group was larger than that in the control group. Regarding the fertilization method, the ICSI rate in the OPTIMUM group was significantly lower than that in the control group, because a significant difference in the prevalence of RIF and RPL was observed between the two groups; however, no significant differences in oocyte retrieval and ET findings were observed.

**TABLE 2:** Assisted reproductive technology and pregnancy outcomes in control and OPTIMUM groups.

	Control *n* = 66	OPTIMUM *n* = 127	*p*‐Value
Oocyte retrieval			
No. of cycles until euploidy detection	1 (1–5)	2 (1–15)	0.84
No. of blastocysts until euploidy detection	2 (1–11)	4 (1–17)	0.02*
Fertilization method			
Conventional IVF	22 (33.3)	66 (52.0)	
Intracytoplasmic sperm injection	44 (66.7)	61 (48.0)	0.02*
Embryo transfer			
Morphologically good blastocyst^a^	56 (84.8)	116 (91.3)	0.22
Culture time until expanded blastocyst^b^			
5 days	43 (65.2)	93 (73.2)	
6 days	23 (34.8)	34 (26.8)	0.25
Frozen–thawed embryo transfer cycle			
Natural cycle	42 (63.6)	78 (61.4)	0.86
Hormonal replacement cycle	24 (36.4)	49 (38.6)	
Personalized embryo transfer^c^	7 (10.6)	25 (19.7)	0.15
Pregnancy outcome			
hCG positive rate	34 (51.5)	101 (79.5)	<0.001*
Clinical pregnancy rate	30 (45.5)	91 (71.7)	<0.001*
Miscarriage rate	4 (13.3)	9 (9.9)	0.73
Live birth rate	26 (39.4)	82 (64.6)	0.001*

*Note*: Data are presented as median (range) or *n* (%).

Abbreviations: OPTIMUM, optimization of thyroid function, thrombophilia, immunity and uterine milieu; RIF, repeated implantation failure; RPL, recurrent pregnancy loss.

^a^ Morphologically good blastocysts are diagnosed as blastocysts except for grade C in both the inner cell mass and the trophectoderm of the Gardner classification.

^b^ Expanded blastocysts are Grade 4 or more of the Gardner classification.

^c^ Personalized embryo transfer was performed based on the results of endometrial receptivity testing including endometrial receptivity analysis (ERA®) and ERPeak^SM^ tests.

* *p* < 0.05.

With regard to pregnancy outcomes, hCG positive rate and clinical pregnancy rate in the OPTIMUM group were significantly higher than those in the control group (hCG positive rate of 79.5% and 51.5% and clinical pregnancy rate of 71.7% and 45.5%, respectively; both *p* < 0.001). No significant difference in miscarriage rate was observed between two groups (9.9% and 13.3%, respectively; *p* = 0.73). Live birth rate in the OPTIMUM group was significantly higher than that in the control group (64.6% and 39.4%, respectively; *p* = 0.001).

**FIGURE 4:**
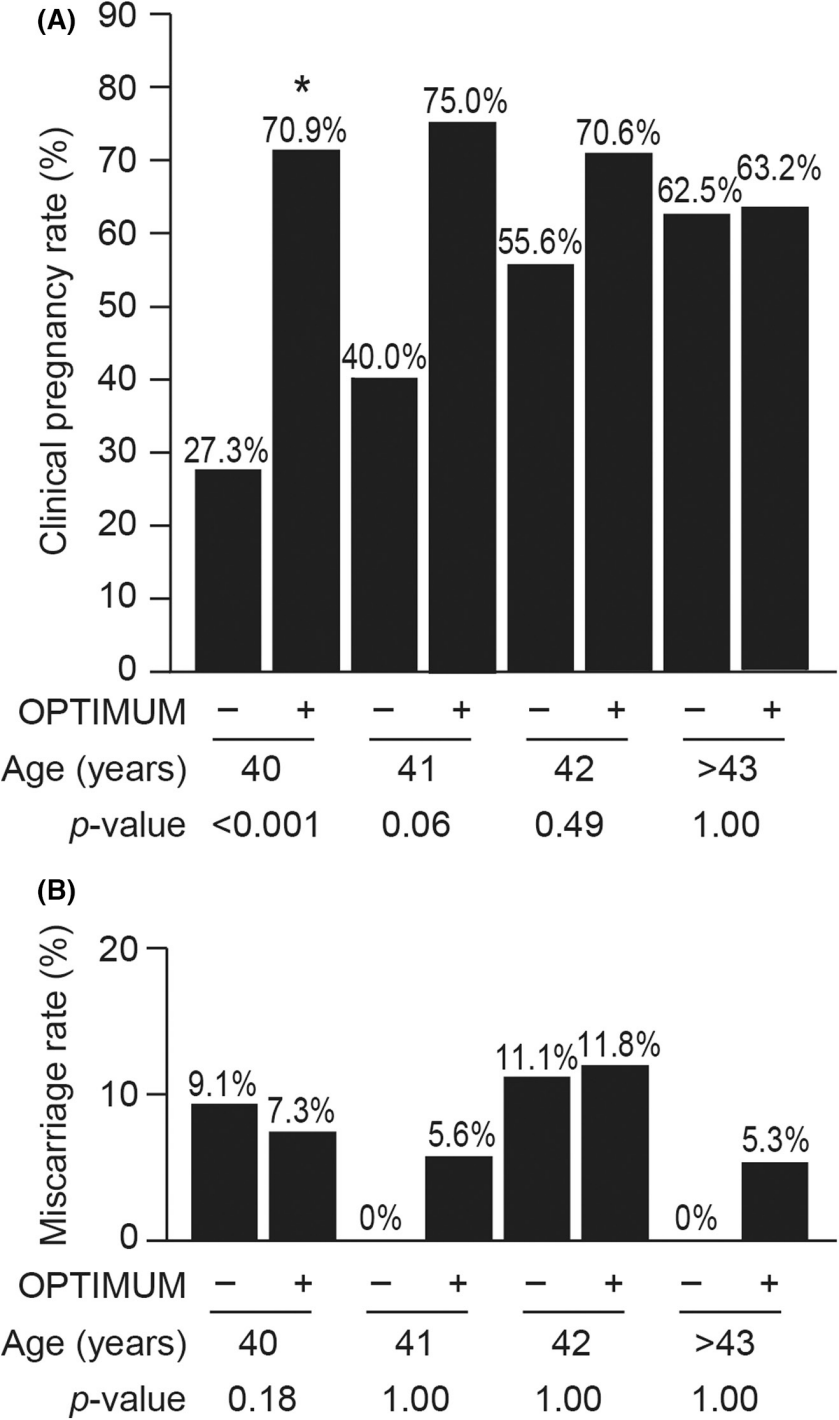
Pregnancy outcomes according to age. Pregnancy outcomes according to age after single euploid blastocyst transfer (SEBT) in the control and OPTIMUM groups are shown. Among women aged 40 years, those who underwent the OPTIMUM treatment strategy had significantly higher clinical pregnancy rates than did those who did not. Women aged 41 years in the OPTIMUM group had also relatively higher clinical pregnancy rate, compared to those in the control; however, no significant differences were noted among women aged ≥42 years. *p < 0.05.

Next, to confirm the age at which the OPTIMUM treatment strategy contributed to pregnancy outcomes in the SEBT cycle, we also analyzed the pregnancy outcomes according to age (Figure 4, Data S1). Accordingly, women aged 40 who received the OPTIMUM treatment strategy had significantly higher clinical pregnancy rate than did those who did not receive such treatment (70.9% and 27.3%, respectively; *p* < 0.001). Women aged 41 years in the OPTIMUM group had also relatively higher clinical pregnancy rate, compared to those in the control (75.0% and 40.0%, respectively; *p* = 0.06). However, no significant differences were noted among women aged ≥42 years. Regarding miscarriage rates, no significant differences were observed among all women over 40.

**FIGURE 5:**
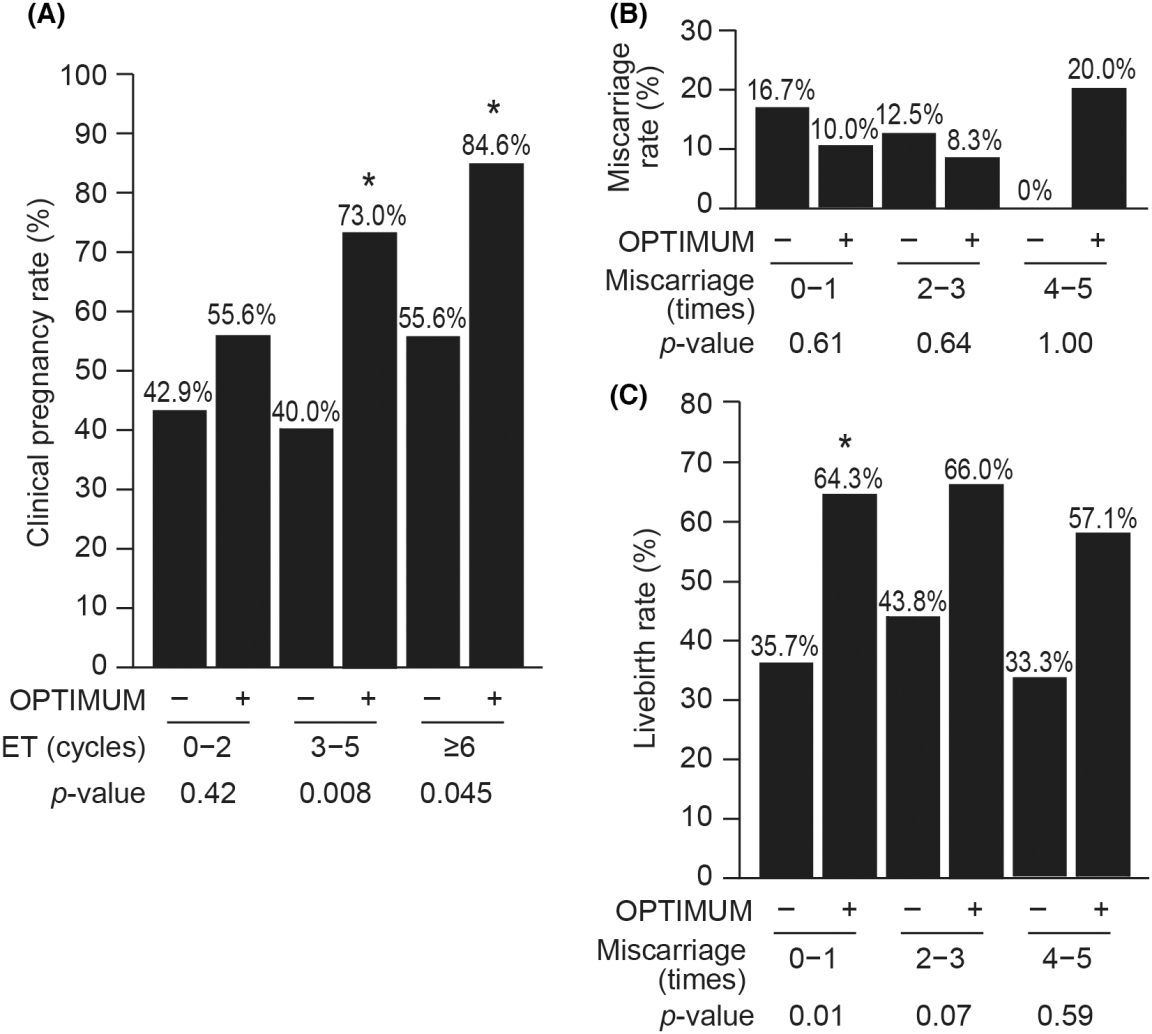
Pregnancy outcomes according to previous embryo transfer cycles and miscarriages. (A) Clinical pregnancy rates according to previous embryo transfer (ET) cycles, (B) Miscarriage rates according to previous number of miscarriages, (C) Livebirth rates according to previous number of miscarriages. Among women with a history of ≥3 ET cycles, those who underwent the OPTIMUM treatment strategy had significantly higher clinical pregnancy rates than did those who did not; however, there were no significant differences in miscarriage rates according to previous number of miscarriages. *p < 0.05.

The pregnancy outcomes according to number of previous ET cycles and miscarriages after SEBT are shown in Figure 5. Among women with a history of 3–5 and ≥6 ET cycles, those who underwent the OPTIMUM treatment strategy had significantly higher clinical pregnancy rates than did those who did not (73.0% and 40.0% for those with 3–5 ET cycles, *p* = 0.008 and 84.6% and 55.6% for those with ≥6 ET cycles, *p* = 0.045, respectively, Figure 3A, Data S2). According to the number of previous miscarriages, relatively higher livebirth rates were observed in the OPTIMUM group compared to those in the control group; however, there were no significant differences of miscarriage rates (Figure 3B,C, Data S3).

### 3.4 Factors predicting livebirth after the OPTIMUM treatment strategy

After the OPTIMUM treatment strategy, 64.6% (82/127 women) of the advanced age women ≥40 years who suffered from multiple instances of reproductive failure were able to bear a child after the first SEBT, whereas the remaining 35.4% experienced implantation failure or miscarriage. To clarify the factors predicting livebirth after the OPTIMUM treatment strategy, we compared the 82 women who had successfully borne a child (success group) and the 45 women who unfortunately experienced implantation failure or pregnancy loss (failure group; Table 3). Regarding endometrial receptivity testing, personalized ET rates based on the results in the success and failure groups were 12.2% (10 women) and 33.3% (15 women), respectively; therefore, this was not included in our analysis (data not shown). Women in the success group had significantly higher rates of morphologically good blastocysts and relatively higher rates of blastocyst cultured for 5 days than did those in the failure group (*p* = 0.02 and 0.06, respectively). No significant differences in age, prevalence of RIF and RPL, and risk factors of implantation failure and pregnancy loss were observed between the groups.

**TABLE 3:** Predictive factors for therapeutic effects of OPTIMUM treatment strategy and single euploidy embryo transfer in the infertile women with live birth.

	Success group^a^ *n* = 82	Failure group^a^ *n* = 45	*p*‐Value	Univariate analysis OR (95% CI)	Multivariate analysis OR (95% CI)
Age, years	41.1 ± 1.3 (40–46)	41.1 ± 1.3 (40–44)	0.93	0.98 (0.74–1.31)	0.93 (0.68–1.28)
Prevalence of RIF	64 (78.0)	32 (71.1)	0.40	1.03 (0.47–2.26)	1.57 (0.34–7.19)
Prevalence of RPL	34 (41.5)	19 (42.2)	1.00	1.06 (0.51–2.23)	1.33 (0.32–5.53)
Embryo quality^b^					
Morphologically good blastocyst	79 (96.3)	37 (82.2)	0.02*	Reference	Reference
Morphologically poor blastocyst	3 (3.7)	8 (17.8)		0.18 (0.04–0.70)	0.26 (0.06–1.13)
Culture time until expanded blastocyst^c^					
5 days	65 (79.3)	28 (62.2)	0.06	Reference	Reference
6 days	17 (20.7)	17 (37.8)		0.43 (0.19–0.96)	0.47 (0.19–1.16)
Risk factors for implantation failure or pregnancy loss					
Impaired intrauterine circumstance	39 (47.6)	29 (64.4)	0.09	0.50 (0.24–1.06)	0.54 (0.24–1.21)
Aberrant high Th1 cell level and/or Th1/Th2 cell ratio	26 (31.7)	12 (26.7)	0.69	1.28 (0.57–2.86)	1.22 (0.51–2.92)
Thyroid disorder	13 (15.9)	6 (13.3)	0.80	1.23 (0.43–3.48)	1.49 (0.46–4.87)
Thrombophilia	13 (15.9)	11 (24.4)	0.25	0.58 (0.24–1.44)	0.76 (0.28–2.05)

*Note*: Data are presented as mean ± SD (range) or *n* (%).

Abbreviations: CI, confidence interval; OR, odds ratio; RIF, repeated implantation failure; RPL, recurrent pregnancy loss.

^a^ To identify the predictive factors for the therapeutic efficacy of the OPTIMUM treatment strategy and single euploidy blastocyst transfer, we compared the 82 women who had successfully childbirth (success group) and the 45 women who ended in implantation failure or pregnancy loss (failure group) in the first single euploidy embryo transfer after the OPTIMUM treatment strategy.

^b^ Morphologically good blastocysts are defined as blastocysts except for grade C in both the inner cell mass and the trophectoderm of the Gardner classification.

^c^ Expanded blastocysts are Grade 4 or more of the Gardner classification.

* *p* < 0.05.

Univariate analysis identified morphologically poor blastocysts (OR = 0.18, 95% CI = 0.04–0.70) and 6 days of culture time until expanded blastocyst (OR = 0.43, 95% CI = 0.19–0.96) as factors predicting unsuccessful childbirth after the OPTIMUM treatment strategy in women with advanced age. However, multivariate analysis failed to identify significant independent predictive factors.

## 4 DISCUSSION

This has been the first report regarding the therapeutic efficacy of combination treatment for abnormalities related to intrauterine circumstances, thyroid function, helper T cells, and thrombophilia on pregnancy outcomes after euploidy ET in the women of advanced age with RIF and/or RPL. In this study, the OPTIMUM treatment strategy promoted clinical pregnancy in 71.7% and childbearing in 64.6% of the women after the first SEBT. The clinical pregnancy and livebirth rates in the OPTIMUM group were significantly higher than those in the control group. In particular, the OPTIMUM treatment strategy improved pregnancy outcomes in the women aged 40 years with/without a history of RIF.

Of various potential causes of implantation failure, except for embryonic factor, evidence‐based treatments for RIF include normalization of intrauterine abnormalities including CE, thyroid dysfunction, and thrombophilia; lifestyle modification; and preimplantation genetic testing for chromosomal abnormalities.^31^ The standard treatment options for RPL include only levothyroxine supplementation for hypothyroidism and low‐dose aspirin and low‐molecular‐weight heparin for antiphospholipid syndrome.^16^ However, treatment for unexplained RPL has not been established.^5^ Consecutive implantation failure and miscarriages can be triggered by multiple factors that decrease the likelihood of embryo implantation or increasing miscarriage rate and not established risk factors for RIF and RPL.^3^, ^6^, ^16^, ^32^ The OPTIMUM treatment strategy targets the representative risk factors for implantation failure and pregnancy loss, including factors for RIF and RPL, thereby promoting childbearing in most of young patients aged <40 years with multiple instances of reproductive failure.^8^, ^9^ Our results showed that the OPTIMUM treatment strategy can contribute to improving pregnancy outcomes in women aged 40 to 41 years or less with a history of RIF and/or RPL.

In our previous studies on the OPTIMUM treatment strategy,^8^, ^9^ the clinical pregnancy rate of single blastocyst transfer after the OPTIMUM in women aged ≥40 years was 42.4% (14/33 ET cycles) in women with RIF, whereas the miscarriage rate after clinical pregnancy was 44.4% (16/36 clinical pregnancies) in women with RPL. The current study showed that after the OPTIMUM treatment strategy and euploid blastocyst transfer, the clinical pregnancy and miscarriage rates were 76.0% (73/96 ET cycles) in women with RIF and 8.1% (3/37 clinical pregnancies) in women with RPL, respectively, which were significantly better than those reported in the previous studies (both *p* < 0.001). Lund, et al.^33^ reported that more than half of women aged ≥40 years with RPL end up without livebirth in 5 years. In our approach, 64.2% (34/53 women) of patients with RPL achieved childbearing after one trial of SEBT, thus the OPTIMUM treatment strategy in combination with PGT‐A is needed for leading to livebirth in advanced age women with RPL. Note that euploid embryo transfer after PGT‐A improves pregnancy outcomes after ET in the advanced age women with RIF and/or RPL; however, the OPTIMUM treatment strategy did not contribute to improving the pregnancy outcomes in the women aged ≥42 years and miscarriage rates in those ≥40 years.

In humans, the incidence of implantation failure and pregnancy loss with embryonic chromosomal abnormalities is considerably high.^1^, ^2^ Therefore, true RIF except for an embryonic factor is uncommon in infertile women who undergo IVF treatment.^34^ The prevalence of RPL is also rare at approximately 2%–5%.^32^, ^35^ True RIF and RPL are expected to be extremely infrequent in women aged ≥40 years with high aneuploidy rate of embryos. In our study, SEBT was performed in the women with RIF after ≥3 ET cycles using blastocysts and RPL ≥2 consecutive clinical pregnancy losses. Based on PGT‐A data from the United States, more than three, four, and eight blastocysts are required when 40‐, 42‐, and 44‐year‐old women aim for one euploid embryo, respectively.^14^ Furthermore, according to a previous report in cytogenic analysis of products of conception, the detection rates of trisomy of the spontaneous abortion in women aged 40–43 years are 89%–95%.^2^ Therefore, RIF women aged ≥42 years and RPL women aged ≥40 years recruited into our study had experienced recurrent reproductive failure mainly due to embryonic chromosomal abnormalities. The OPTIMUM contributed to the increased clinical pregnancy rates in RIF women aged 40–41 years, but not miscarriage rates. Univariate analysis to identify factors predicting livebirth after the OPTIMUM treatment strategy demonstrated that low‐quality embryos were involved in poor pregnancy outcomes; however, multivariate analysis did not detect any predictive factors. This suggests that embryonic factors are most important for successful livebirth in women of advanced age.

Repeated experience of implantation failure, pregnancy loss, and stillbirth can frustrate patients and induce feelings of despair, self‐dispraise, and impotence, which can lead to depression, anxiety disorders, and insomnia.^36^, ^37^, ^38^ Chronic psychological stress has been linked to increased risk of infertility and pregnancy loss.^26^, ^27^, ^39^, ^40^, ^41^ Stress‐induced anxiety induces an increase in the Th1/Th2 cell ratio with Th1 bias.^42^ In fact, our previous report found that four or more occurrences of implantation failure after ET and two or more pregnancy losses were associated with aberrant high Th1 cell levels and Th1/Th2 cell ratios.^22^ In our study, the OPTIMUM treatment strategy contributed to increased livebirth rates after SEBT; therefore, assistance in childbearing including the OPTIMUM and PGT‐A should be discussed with patients before their fecundity is decreased with female aging and immunological rejection by recurrent reproductive failure.

Many patients with RIF and RPL sacrifice exorbitant amounts of money and risk harming themselves to experience childbearing.^43^ Our RIF/RPL testing in the OPTIMUM treatment strategy does not include expensive examinations, such as ERA and endometrial microbiome testing; thus, this treatment strategy would cost around 50 000–60 000 yen ($US 455–545).^8^ In addition, the medical insurance in Japan covers a portion of these examinations, including testing for thrombophilia and thyroid function in women with a history of two consecutive pregnancy losses. To obtain one euploid embryo, some of our patients underwent ten or more cycles of oocyte retrieval and shouldered the expensive medical costs for PGT‐A. In patients over 42 years of age with RIF or RPL, the OPTIMUM treatment strategy may be excessive, yet they do not need to prepare a large sum of money for it. The OPTIMUM treatment strategy can be performed in patients who wish to undergo it.

The current study has some limitations worth noting. First, this was an observational study, and the patients in the control group were not offered or did not desire RIF/RPL testing, which could potentially introduce selection bias. In the OPTIMUM group, the interventions may have led to the improved pregnancy outcomes as tender loving care.^44^ Second, no global standard diagnostic criteria for CE have yet been established. As such, we defined CE as the presence of ≥5 CD138‐positive cells in 10 nonoverlapping random stromal areas. However, differences in criteria might lead to different outcomes.

In conclusion, this has been the first report on the OPTIMUM treatment strategy for pregnancy outcomes after SEBT in advanced age women with a history of RIF/RPL. Notably, our findings showed that the OPTIMUM treatment strategy increased clinical pregnancy rates after ET using euploid embryos among women aged ≤40 to 41 years or a history of RIF. However, PGT‐A with and without the OPTIMUM treatment strategy resulted in low miscarriage rates, with the OPTIMUM treatment strategy failing to contribute to the prevention of miscarriages. Embryonic chromosomal abnormality is the main cause of recurrent reproductive failure in women aged ≥42 years; therefore PGT‐A is needed, whereas the OPTIMUM treatment strategy may be excessive. However, in women aged 40 to 41 years or less and a history of RIF, testing and treatment for both embryonic and non‐embryonic factors using PGT‐A and the OPTIMUM treatment strategy are needed for shortening the time to livebirth.

## CONFLICT OF INTEREST STATEMENT

All authors have no conflicts of interest to declare, relevant to this study. Tetsuo Maruyama is an Editorial Board member of Reproductive Medicine and Biology and a co‐author of this article. To minimize bias, they were excluded from all editorial decision‐making related to the acceptance of this article for publication. Human rights statement and informed consent: This study was approved by the local ethics committee of Sugiyama Clinic (No. 18–002 and 22–008). All procedures followed were in accordance with the ethical standards of the responsible committee on human experimentation and with the Helsinki Declaration of 1964 and its later amendments. The data that support the findings of this study are available on request from the corresponding author.

## Supporting information


Data S1–S3.

Table S1.
