# Supplementary material for: Therapeutic efficacy of the optimization of thyroid function, thrombophilia, immunity and uterine milieu (OPTIMUM) treatment strategy on pregnancy outcomes after single euploid blastocyst transfer in advanced age women with recurrent reproductive failure
Source: Reprod Med Biol. 2023 Dec 22;22(1):e12554. doi: 10.1002/rmb2.12554 (PMC10739138; doi:10.1002/rmb2.12554)
Supplement: Supplementary file 1 — Data S1–S3. [file RMB2-22-e12554-s001.docx]

**Data S1. Pregnancy outcome according to age after single euploid blastocyst transfer**

| Age (years) | 40 | | | 41 | | | 42 | | | 43 | | |
| --- | --- | --- | --- | --- | --- | --- | --- | --- | --- | --- | --- | --- |
|  | Control  n = 22 | OPTIMUM  n = 55 | *p* value | Control  n = 10 | OPTIMUM  n = 36 | *p* value | Control  n = 18 | OPTIMUM  n = 17 | *p* value | Control  n = 16 | OPTIMUM  n = 19 | *p* value |
| Clinical pregnancy rate | 6 (27.3) | 39 (70.9) | <0.001 | 4 (40.0) | 27 (75.0) | 0.06 | 10 (55.6) | 12 (70.6) | 0.49 | 10 (62.5) | 12 (63.2) | 1.00 |
| Miscarriage rate | 2 (9.1) | 4 (7.3) | 0.18 | 0 (0) | 2 (5.6) | 1.00 | 2 (11.1) | 2 (11.8) | 1.00 | 0 (0) | 1 (5.3) | 1.00 |
| Live birth rate | 4 (18.2) | 35 (63.6) | <0.001 | 4 (40.0) | 25 (69.4) | 0.14 | 8 (44.4) | 10 (58.8) | 0.51 | 10 (62.5) | 11 (57.9) | 1.00 |

**Data S2. Pregnancy outcome according to number of previous embryo transfer cycles after single euploid blastocyst transfer**

| Embryo transfer (cycles) | 0 | | | 1 | | | 2 | | | 3 | | |
| --- | --- | --- | --- | --- | --- | --- | --- | --- | --- | --- | --- | --- |
|  | Control  n = 12 | OPTIMUM  n = 17 | *p* value | Control  n = 6 | OPTIMUM  n = 4 | *p* value | Control  n = 10 | OPTIMUM  n = 6 | *p* value | Control  n = 12 | OPTIMUM  n = 32 | *p* value |
| Clinical pregnancy rate | 4 (33.3) | 11 (64.7) | 0.14 | 4 (66.7) | 2 (50.0) | 1.00 | 4 (40.0) | 2 (33.3) | 1.00 | 4 (33.3) | 25 (78.1) | 0.01 |
| Miscarriage rate | 0 (0) | 0 (0) | 1.00 | 0 (0) | 0 (0) | 1.00 | 0 (0) | 0 (0) | 1.00 | 2 (50.0) | 3 (12.0) | 0.17 |
| Live birth rate | 4 (33.3) | 11 (64.7) | 0.14 | 4 (66.7) | 2 (50.0) | 1.00 | 4 (40.0) | 2 (33.3) | 1.00 | 2 (16.7) | 22 (68.8) | 0.005 |

| Embryo transfer (cycles) | 4 | | | 5 | | | 6 | | | >7 | | |
| --- | --- | --- | --- | --- | --- | --- | --- | --- | --- | --- | --- | --- |
|  | Control  n = 8 | OPTIMUM  n = 25 | *p* value | Control  n = 0 | OPTIMUM  n = 17 | *p* value | Control  n = 6 | OPTIMUM  n = 5 | *p* value | Control  n = 12 | OPTIMUM  n = 21 | *p* value |
| Clinical pregnancy rate | 4 (50.0) | 18 (72.0) | 0.39 | – | 11 (64.7) | – | 4 (66.7) | 4 (80.0) | 1.00 | 6 (50.0) | 18 (85.7) | 0.04 |
| Miscarriage rate | 0 (0) | 3 (16.7) | 1.00 | – | 3 (27.3) | – | 0 (0) | 0 (0) | 1.00 | 2 (33.3) | 0 (0) | 0.054 |
| Live birth rate | 4 (50.0) | 15 (60.0) | 0.70 | – | 8 (47.1) | – | 4 (66.7) | 4 (80.0) | 1.00 | 4 (33.3) | 18 (85.7) | 0.005 |

**Data S3. Pregnancy outcome according to number of previous miscarriages after single euploid blastocyst transfer**

| Miscarriage (times) | 0 | | | 1 | | | 2 | | | 3 | | |
| --- | --- | --- | --- | --- | --- | --- | --- | --- | --- | --- | --- | --- |
|  | Control  n = 16 | OPTIMUM  n = 43 | *p* value | Control  n = 12 | OPTIMUM  n = 27 | *p* value | Control  n = 16 | OPTIMUM  n = 35 | *p* value | Control  n = 16 | OPTIMUM  n = 15 | *p* value |
| Clinical pregnancy rate | 4 (25.0) | 29 (67.4) | 0.007 | 8 (66.7) | 21 (77.8) | 0.69 | 10 (62.5) | 24 (68.6) | 0.75 | 6 (37.5) | 12 (80.0) | 0.03 |
| Miscarriage rate | 2 (50.0) | 3 (10.3) | 0.10 | 0 (0) | 2 (9.5) | 1.00 | 0 (0) | 2 (8.3) | 1.00 | 2 (33.3) | 1 (8.3) | 0.25 |
| Live birth rate | 2 (12.5) | 26 (60.5) | 0.001 | 8 (66.7) | 19 (70.4) | 1.00 | 10 (62.5) | 22 (62.9) | 1.00 | 4 (25.0) | 11 (73.3) | 0.01 |

| Miscarriage (times) | 4 | | | 5 | | |
| --- | --- | --- | --- | --- | --- | --- |
|  | Control  n = 6 | OPTIMUM  n = 4 | *p* value | Control  n = 0 | OPTIMUM  n = 3 | *p* value |
| Clinical pregnancy rate | 2 (33.3) | 3 (75.0) | 0.52 | – | 2 (66.7) | – |
| Miscarriage rate | 0 (0) | 1 (33.3) | 1.00 | – | 0 (0) | – |
| Live birth rate | 2 (33.3) | 2 (50.0) | 1.00 | – | 2 (66.7) | – |
