# Supplementary material for: Therapeutic efficacy of the optimization of thyroid function, thrombophilia, immunity and uterine milieu (OPTIMUM) treatment strategy on pregnancy outcomes after single euploid blastocyst transfer in advanced age women with recurrent reproductive failure
Source: Reprod Med Biol. 2023 Dec 22;22(1):e12554. doi: 10.1002/rmb2.12554 (PMC10739138; doi:10.1002/rmb2.12554)
Supplement: Supplementary file 2 — Table S1. [file RMB2-22-e12554-s002.docx]

**Supplementary table 1. Prevalence of risk factors for implantation failure and pregnancy loss**

|  | **RIF, n = 74** | **RPL, n = 31** | **RIF+RPL, n = 22** | **Total, n = 127** |
| --- | --- | --- | --- | --- |
| **Intrauterine circumstance**  Normal  Chronic endometritis  Endometrial polyp  Intrauterine adhesion  Submucosal myoma  Septate uterus  **Total women with impaired intrauterine circumstance**^†^ | 32 (43.2)  35 (47.3)  6 (8.1)  1 (1.4)  2 (2.7)  1 (1.4)  **42 (56.8)** | 16 (51.6)  13 (41.9)  5 (16.1)  0 (0)  0 (0)  1 (3.2)  **15 (48.4)** | 11 (50.0)  10 (45.5)  2 (9.1)  1 (4.5)  0 (0)  0 (0)  **11 (50.0)** | 59 (46.5)  58 (45.7)  13 (10.2)  2 (1.6)  2 (1.6)  2 (1.6)  **68 (53.5)** |
| **Immunological tolerance**  **Vitamin D**^‡^  25-hydroxyvitamin D3, ng/mL, mean ± SD  Sufficiency  Insufficiency  Deficiency  **Total women with lack of vitamin D** | 25.6 ± 10.4  31 (41.9)  33 (44.6)  10 (13.5)  **43 (58.1)** | 22.2 ± 7.6  4 (12.9)  24 (77.4)  3 (9.7)  **27 (87.1)** | 32.8 ± 21.4  11 (50.0)  11 (50.0)  0 (0)  **11 (50.0)** | 26.2 ± 12.8  46 (36.2)  68 (53.5)  13 (10.2)  **81 (63.8)** |
| **Helper T cells**  Th1 cell, %  Th2 cell, %  Th1/Th2 cell ratio  **Total women with aberrant high Th1 and/or Th1/Th2 cell ratio** | 20.8 ± 6.9  2.4 ± 1.0  9.6 ± 4.6  **20 (27.0)** | 20.4 ± 6.8  2.8 ± 1.3  9.0 ± 5.8  **8 (25.8)** | 24.2 ± 9.7  3.0 ± 1.5  10.8 ± 5.3  **10 (45.5)** | 21.7 ± 7.4  2.6 ± 1.2  9.7 ± 5.0  **38 (29.9)** |
| **Thyroid function**  Subclinical hypothyroidism  Overt hypothyroidism  Hyperthyroidism  Thyroid peroxidase antibody-positive^§^  Thyroid cancer  **Total women with thyroid dysfunction** | 10 (13.5)  0 (0)  1 (1.4)  7 (9.5)  1 (1.4)  **13 (17.6)** | 1 (3.2)  1 (3.2)  0 (0)  1 (3.2)  0 (0)  **2 (6.5)** | 4 (18.2)  0 (0)  0 (0)  3 (13.6)  0 (0)  **4 (18.2)** | 15 (11.8)  1 (0.8)  1 (0.8)  11 (8.7)  1 (0.8)  **19 (15.0)** |
| **Thrombophilia**  Lupus anticoagulant positive  Anticardiolipin antibody (IgG, IgM) positive  Anti-β2-GP1 antibody (IgG, IgM) positive  Protein C deficiency  Protein S deficiency  Factor XII deficiency  **Total women with thrombophilia**^¶^ | 1 (1.4)  5 (6.8)  1 (1.4)  0 (0)  5 (6.8)  3 (4.1)  **14 (18.9)** | 0 (0)  0 (0)  0 (0)  1 (3.2)  3 (9.7)  1 (3.2)  **5 (16.1)** | 1 (4.5)  1 (4.5)  0 (0)  0 (0)  1 (4.5)  2 (9.1)  **5 (22.7)** | 2 (1.6)  6 (4.7)  1 (0.8)  1 (0.8)  9 (7.1)  6 (4.7)  **24 (18.9)** |
| **No risk factor for RIF and RPL** | **20 (27.0)** | **8 (25.8)** | **3 (13.6)** | **31 (24.4)** |

Note: Data are presented as n (%) or mean ± SD. RIF = repeated implantation failure; RPL, recurrent pregnancy loss.

^†^ Nine women with intrauterine disorders had chronic endometritis.

^‡^ Vitamin D deficiency, insufficiency and sufficiency were diagnosed as 25-hydroxyvitamin D_3_ levels of <12, between ≥12 and <30, and ≥30 ng/mL, respectively.

^§^ Ten women with thyroid peroxidase antibody-positive included 1 and 9 women with overt and subclinical hypothyroidism, respectively.

^¶^ One woman had two thrombophilia factors.
